# Nostrums

**Published:** 1880-05

**Authors:** 


### EXCERPTA.

NOSTRUMS.—The manufacturers of such copyrighted medicines as physicians are asked to prescribe object very much to the application of the name of "nostrum" to their wares. They not only do not object to the application of the name to Perry Davis' Pain Killer, or Jaynes' Expectorant, or the Golden Medical Discovery, or any other of the thousand and one preparations which are advertised in flaming posters on fences and dead walls, or which take up so much space in the secular prints, but they highly commend its use in connection with these. When, however, it is applied to their compounds—medicines bearing names evolved from the inventive faculty of their own brains, and recognized in neither the pharmacopœias nor botanies of their own or any other land—they protest. Let us enquire whether or not this application of the term be correct. It is only denied by these makers that the goods on which they hold the exclusive right of manufacture cannot, because of the protection which the law affords in virtue of the copyright, be made by any other manufacturer. These medicines are, in point of fact theirs. In speaking of them themselves they call them "ours," and no one dares infringe on their ownership of them, any more than he dares take the goods or chattels of another without a consideration or without their permission. Now this is all that is implied in the word "nostrum." A nostrum is not necessarily a secret medicine, but it is always a proprietary one, the word being from the Latin *noster*, meaning *ours*. Wherein, then, lies the difference?

These manufacturers assert the fact that they publish the formulæ of their mixtures as a reason why these mixtures should not have applied to them a name which has become opprobrious. But do not the manufacturers of the most notorious patent medicines also publish their formulæ? By consulting the records of the patent office at Washington, the formulæ of all of them may be procured, but the letters patent (from *pateo*, to open), while permitting their opening for the persual of all who may desire, at the same time prohibits the appropriation of any of the ideas for which the letters were granted. While the proprietor of a copyrighted medicine asserts truly that his formula is published, he knows that it is equally true that this publication avails the profession nothing. The instant in which he might detect another manufacturer compounding a mixture from this formula and attaching to it the name he has given it he would seek the protection which the law provides.

It has always been a mystery to us how physicians, inordinate sticklers for the ethics of the profession as enunciated by the code, should support by their patronage pharmacists who by copyrighting their preparations, live in open and flagrant violation of this code. Consistency, however, is a jewel, which some very estimable men seem never to have possessed themselves of.

